# Impact of Glutinous Rice Varieties from Different Regions on Microbial Community Structure, Metabolic Profiles, and Flavor Characteristics of Chinese Rice Wine (Huangjiu)

**DOI:** 10.3390/foods14071261

**Published:** 2025-04-03

**Authors:** Qi Peng, Linyuan Li, Guangfa Xie

**Affiliations:** 1 National Engineering Research Center for Chinese CRW (Branch Center), School of Life and Environmental Sciences, Shaoxing University, Shaoxing 312000, China; dr_pengqi@163.com (Q.P.); lilinyuan2001@163.com (L.L.); 2 Key Laboratory of Pollution Exposure and Health Intervention of Zhejiang Province, College of Biology and Environmental Engineering, Zhejiang Shuren University, Hangzhou 310015, China

**Keywords:** Huangjiu, glutinous rice varieties, microbial diversity, metabolomic profiling, flavor composition, regional fermentation

## Abstract

Huangjiu is a traditional alcoholic beverage in China, but because of the differences in fermentation conditions and raw materials, how to optimize the flavor quality of Huangjiu is facing challenges. This study used high-throughput sequencing (HTS) to investigate microbial diversity in Huangjiu brewed from glutinous rice from five regions in China. Metabolic pathway annotation, electronic senses, and metabolite analysis elucidated the relationships between rice variety, microbial communities, flavor profiles, and metabolic characteristics of Huangjiu. Statistically significant differences in microbial community structure and flavor profiles were observed across Huangjiu samples (*p* < 0.05), with ten dominant microbial genera identified. Lactic acid bacteria (LAB) enriched in Guizhou and Hubei were positively correlated with higher organic acid (12.36 and 12.30 mg/mL, respectively) and lower amino acid levels (2985 and 2920 mg/L, respectively), contributing to a more pronounced sourness in these Huangjiu. Conversely, Huangjiu from Zhejiang, Guangxi, and Jilin exhibited higher concentrations of *Saccharopolyspora*, *Saccharomonospora*, *Saccharomyces*, and *Bacillus*, associated with elevated amino acid (3706, 3695, and 3700 mg/L, respectively) and reduced organic acid levels (10.11, 9.92 and 10.10 mg/mL, respectively), resulting in sweetness and bitterness. These findings provide valuable insights for optimizing Huangjiu flavor and quality through targeted microbial and fermentation management.

## 1. Introduction

Huangjiu is a traditional Chinese fermented alcoholic drink, known for its distinctive yellow color, sweet aroma, and rich nutritional value. The fermentation process of Huangjiu involves a combination of rice, qu (a fermentation starter), and yeast, which work together in mixed fermentation [1]. The brewing process begins with soaking and steaming the grains, followed by the addition of wheat Qu and yeast, which are thoroughly mixed to form the Huangjiu fermentation mash. This mash then undergoes simultaneous saccharification and alcoholic fermentation. During saccharification, molds in wheat Qu hydrolyze starch from the grains into glucose, while in the alcoholic fermentation phase, *Saccharomyces cerevisiae*—a yeast strain distinct from baker’s yeast, known for its high ethanol tolerance—ferments the glucose into ethanol. After fermentation is complete, the mash is pressed, clarified, and sterilized, and the resulting liquid is sealed in vats for aging to enhance its depth and complexity.

Huangjiu is categorized into several varieties based on raw materials, Qu types, and fermentation techniques. The main types include glutinous rice Huangjiu, millet Huangjiu, indica rice Huangjiu, and red yeast Huangjiu, each characterized by unique fermentation processes and distinct sensory attributes. Variations in raw materials, fermentation starters, and techniques lead to considerable diversity in both the flavor profiles and microbial communities of Huangjiu. These differences present challenges for standardizing Huangjiu production while maintaining consistent flavor and quality. Chen et al. conducted microbiome analysis on three traditional starter cultures (CMQ, NBQ, and YCQ) and found that there were significant differences in microbial communities among different starter cultures [2]. For example, *Pantoea* and *Rhizopus* were dominant in CMQ. NBQ and YCQ are dominated by *Pediococcus*, *Lactobacillus*, and *Candida*. These differences lead to differences in the volatile flavor components of Hunagjiu, among which NBQ Hunagjiu shows better sensory quality due to abundant volatile components (such as phenylethanol and ethyl acetate). Chen et al. [3] found that the core microorganisms in JIUYAO (such as Pediococcus, Weissella, Saccharomycopsis, and Rhizopus) significantly affect saccharification, acid production, and alcohol generation, and are closely related to several volatile flavor compounds (such as phenylethanol, ethyl butyrate, and ethyl propionate). These microorganisms play important roles in the fermentation and flavor formation of Shaoxing-jiu and provide a theoretical basis for the development of new artificial starters. Despite the progress in understanding the effects of microbial composition in fermentation starters (qu and JIUYAO) on Huangjiu, there has been limited exploration of the influence of grains, the primary raw material, microbial community structure, metabolic characteristics, and flavor profiles.

Beyond the microbial composition of fermentation starters (Qu and Jiuyao), the selection of grains, as the primary raw material for Huangjiu brewing, also has the potential to significantly influence its characteristics. Shen et al. conducted an in-depth study on the effects of different grains (buckwheat, sorghum, indica rice, glutinous rice, and black rice) on the physicochemical properties, volatile compounds, and microbial communities in Chinese Huangjiu. Among these, buckwheat Huangjiu exhibited the highest abundance of Weissella, a genus positively associated with aroma formation. Additionally, buckwheat Huangjiu demonstrated the fastest reduction in reducing sugars and had the highest concentrations of esters, alcohols, phenolic acids, and amino acids, leading to superior sensory ratings, particularly in honey, floral, creamy, and umami attributes [4]. Comparisons between glutinous rice and indica rice-based Huangjiu have revealed significant differences in the composition and concentration of key flavor compounds (e.g., esters, alcohols, and acids), as well as variations in sensory attributes such as bitterness, astringency, ester aroma, and overall liquor aroma. These differences are likely attributable to intrinsic grain properties, including protein content and starch structure [5]. These findings collectively indicate that the microbial composition, sensory characteristics, and flavor compound profiles of Huangjiu are strongly influenced by grain selection, ultimately affecting overall product quality. Among these raw materials, glutinous rice, with its high amylopectin content, facilitates efficient saccharification and fermentation, making it the preferred grain for traditional aromatic Huangjiu production [6]. However, the relationship between glutinous rice varieties from different regions and their impact on Huangjiu production remains poorly understood.

High-throughput sequencing has emerged as a valuable method for investigating microbial diversity in fermentation starters, revealing significant variations in microbial communities. For example, analysis of microbial composition in six distinct starters showed that microbial diversity plays a crucial role in determining the flavor and quality of Huangjiu [7]. Likewise, sequencing of bacterial 16S rRNA genes and fungal internal transcribed spacer II (ITS2) regions in Huangjiu has highlighted the profound influence of microbial starters on both metabolic processes and sensory attributes [8]. Such research has enhanced our understanding of how microbial communities contribute to the unique characteristics of Huangjiu. Electronic sensor technology (Electronic Nose and Electronic Tongue), which can simulate human senses and accurately evaluate the flavor profile (aroma and taste) of food, has been widely used in the determination of food’s sensory attributes [9].

In this study, we address this gap by investigating the effects of glutinous rice varieties from five regions in China on the microbial community structure, metabolic characteristics, and flavor profiles of Huangjiu. Using HTS, we evaluated the microbial diversity and metabolic pathways in 50 Huangjiu samples. Additionally, we applied electronic sensor technology to assess the flavor profiles of the samples and employed high-performance liquid chromatography (HPLC) and an automatic amino acid analyzer to quantify organic acids and amino acids. Furthermore, we explored the correlations between dominant microbial genera and the key organic acid/amino acid metabolites. These findings will provide critical insights for improving Huangjiu production by optimizing the selection of glutinous rice varieties and microbial fermentation processes to enhance quality and consistency.

## 2. Materials and Methods

### 2.1. Materials, Reagent and Instruments

Materials: glutinous rice: bought from Shaoxing local market, China; Jianhu water: coming from Shaoxing Kuaiji Mountain, China; wheat qu: Shaoxing Guyue Longshan company, China; saccharifying enzyme: 100,000 U/g, Angel Enzyme Preparation (Yichang) Co., Ltd., China; highly active dry yeast: Angel Enzyme Preparation (Yichang) Co., Ltd., China.

Reagent: 3, 5-dinitrosalicylic acid (analytically pure): Tianjin Guangfu Fine Chemical Research Institute; C18 reverse-phase column (4.6 mm × 250 mm, 5 μm); all organic acids standard solution are purchased from Sigma Aldrich (Shanghai) Trading Co., Ltd., China; Amino acid mixture standard solution (phosphoserine, taurine, phosphoethanolamine, urea, aspartic acid, hydroxyproline, threonine, serine, aspartamide, glutamic acid, α-aminoadiac, proline, glycine, alanine, citrulline, A-aminobutyric acid, valine, cystine, methionine, isoleucine, leucine, tyrosine, phenylalanine, β-alanine, β-aminoisobutyric acid, Y-aminobutyric acid, histidine, 3-methylhistidine, 1-methylhistidine, carnosine, tryptophan, ornithine, lysine, arginine, sulfoalanine, methionine sulfone, all at 2.5 umol/mL), sample diluent and Ninhydrin (Sykam (Beijing) Scientific Instrument Co., Ltd., China); Fast DNA SPIN Kit for Soil (Beijing Bitbo Biotechnology Co., Ltd., China); NanoDrop 2000 spectrophotometer (Baidaoheng Instrument Equipment (Beijing) Co., Ltd., China); AMPure XP system (Shanghai Rui An Biotechnology Co., Ltd., China); AMPure XP system (Shanghai Rui An Biotechnology Co., Ltd., China).

Instruments: EL204 Electronic Balance, METTler Toledo Technology (Shanghai) Co., Ltd., China; PHSJ-4FpH meter, Shanghai Yi Electrical Scientific Instrument Co., Ltd., China; GIBER-TINI QUICK Wine analyzer, Suzhou Sainz Instrument Co., Ltd., China; HH6 digital constant temperature water-bath pot, Changzhou Ronghua Instrument Manufacturing Co., Ltd., China; DHG-9240A type a thermostatic drum wind drying oven, Shanghai Yiheng Scientific Instrument Co., Ltd., China; Surface Acoustic Wave (SAW)-based E-nose (zNose4300, Beijing Huayi Tongtai Environmental protection Technology Co., Ltd., China), TS-5000Z E-tougue system (Beijing Yingsheng Hengtai Technology Co., Ltd., China); high-performance liquid chromatography (HPLC) system LC-2010, Dingxin Jingke (Beijing) Instrument Co., Ltd., China; L-8900; automatic amino acid analyzer (Hitachi Scientific Instruments (Beijing) Co., Ltd., China); Qubit 2.0 Fluorometer (Thermo Fisher Technology (Shanghai) Co., Ltd., China); Illumina MiSeq platform (Illumina, San Diego, CA, USA).

### 2.2. Sample Collection

Glutinous rice for fermented Huangjiu was gathered from five representative provinces in different locations of China, including Zhejiang in the east, Guangxi in the south, Hubei in the central, Guizhou in the southwestern, and Jinlin in northeast China. Glutinous rice used for Huangjiu fermentation was sourced from five representative provinces across different regions of China, namely Zhejiang (east), Guangxi (south), Hubei (central), Guizhou (southwest), and Jilin (northeast). These regions represent distinct climatic zones, each with unique agricultural practices, cultivation systems, and other agronomic attributes, as detailed in Table 1.

At the same time, a uniform brewing process and a consistent set of auxiliary materials were employed across all batches. The materials used include cooked glutinous rice, Jianhu water, cooked wheat qu, raw wheat qu, highly active dry yeast, and a saccharifying enzyme. Each of these ingredients plays a distinct role in the fermentation process of Huangjiu. Cooked glutinous rice serves as the primary source of starch. During saccharification, the starch is enzymatically converted into dextrins and oligosaccharides, which contribute to a fuller and more mellow mouthfeel in the final product. The water used is Jianhu water, traditionally regarded as optimal for Huangjiu fermentation due to its mineral content. This water is characterized by its clarity, high dissolved oxygen, low color, high transparency, and low oxygen consumption, making it ideal for brewing. Wheat koji, produced from wheat, acts as a crucial saccharification agent. It contains various molds and saccharifying microorganisms that convert the starch in glutinous rice into fermentable sugars, thereby providing essential nutrients for the growth and metabolic activity of other microorganisms during fermentation. Two types of wheat koji are used: cooked wheat koji: made by mixing steamed wheat koji with a pure culture of saccharifying microorganisms, which provides robust saccharification due to the controlled propagation conditions; however, the limited microbial diversity may restrict the complexity of flavor formation. Cooked wheat koji: made by mixing steamed wheat koji with a pure culture of saccharifying microorganisms, which provides robust saccharification due to the controlled propagation conditions; however, the limited microbial diversity may restrict the complexity of flavor formation. Raw wheat koji: Prepared through natural fermentation, it contains a rich diversity of cells, molds, and yeasts, which actively degrade the raw materials and generate a variety of nutritional and flavor compounds. The combined use of cooked and raw wheat koji helps maintain a highly active saccharification metabolism and preserves a richer flavor profile. The highly active dry yeast is a specialized brewing yeast (*Saccharomyces cerevisiae*) known for its high ethanol tolerance, enabling it to efficiently ferment the sugars derived from the starch into alcohol. In addition, the saccharifying enzyme, also known as glucoamylase, is sourced from Angel Enzyme Preparation (Yichang) Co., Ltd., China. This enzyme hydrolyzes the α-1,4 and α-1,6 glycosidic bonds at the non-reducing ends of starch molecules, converting them into glucose. Its use not only reduces the amount of wheat koji required but also prevents overactive yeast fermentation that can lead to premature yeast senescence.

The samples of Huangjiu brewed from glutinous rice in different production areas are hereinafter referred to as Guizhou Huangjiu, Hubei Huangjiu, Zhejiang Huangjiu, Guangxi Huangjiu, and Jilin Huangjiu. The wine sample was fermented for 70 days and the indicators of them meet the national yellow rice wine standard GB/T 13662-2018 [10].

### 2.3. Huangjiu Brewing Scheme

The brewing process of Huangjiu was standardized to ensure consistency across samples and isolate the impact of glutinous rice varieties from different regions. The proportions of raw materials used for brewing were strictly followed according to the formulation provided in Table 2, adhering to established brewing protocols for Chinese rice wine production. This standardization allowed us to attribute variations in microbial community structure, metabolic profiles, and flavor characteristics primarily to the differences in glutinous rice varieties.

All raw materials, including glutinous rice, cooked wheat qu, raw wheat qu, highly active dry yeast, and saccharifying enzyme, were sourced from a single supplier to ensure uniformity across the batches. The fermentation process was conducted in a temperature-controlled environment to mimic traditional fermentation conditions while ensuring experimental reproducibility. The brewing process followed a standard sequence: (1) soaking and steaming the glutinous rice, (2) inoculating the rice with prepared qu and yeast, (3) adding a saccharifying enzyme to enhance the breakdown of starches into fermentable sugars, and (4) allowing the mixture to ferment for 70 days at 18–25 °C. At the end of the 70-day fermentation period, the wine samples were filtered to remove solid residues, and the liquid portion was collected for subsequent microbial, metabolic, and sensory analyses. The fermented Huangjiu samples were immediately stored at −20 °C to preserve their biochemical integrity until further analysis.

### 2.4. Determination of Physicochemical Parameters

The content of reducing sugar was determined by 3, 5-dinitrosalicylic acid colorimetry. The alcohol content was determined according to the method in GB 5009.225-2016 [11]. PH value was determined according to the method in GB/T 13662-2018 “Huangjiu—General Requirements”. The temperature was measured with a Thermoprobe TL-1A thermometer from Xiamen Jielian Instrument Equipment Technology Co., Ltd., China.

In order to ensure that the change in physicochemical parameters is only decided by the fermentation process, the external fermentation conditions such as temperature and fermentation time of all Huangjiu are standardized before the formal fermentation begins. Fermentation temperature was maintained at 18–25 °C throughout the process. Fermentation duration was standardized to 70 days, ensuring that all samples underwent an identical aging period.

### 2.5. DNA Extraction

Genomic DNA was extracted from the Huangjiu samples using the Fast DNA SPIN Kit for Soil (Beijing Bitbo Biotechnology Co., Ltd., China), following the manufacturer’s protocol. Approximately 0.5 g of each Huangjiu sample was used for DNA extraction. The samples were homogenized thoroughly to disrupt microbial cell walls. Bead-beating was applied to mechanically lyse the cells, while chemical reagents facilitated the release of genomic DNA into the solution. The extracted DNA was then purified using a series of spin filters provided in the kit. The purity and concentration of the extracted DNA were evaluated using a NanoDrop 2000 spectrophotometer (Baidaoheng Instrument Equipment (Beijing) Co., Ltd., China), with an optimal A260/A280 ratio between 1.8 and 2.0 indicating high-purity DNA suitable for downstream applications such as high-throughput sequencing. Additionally, DNA integrity was assessed by agarose gel electrophoresis. The extracted DNA was subsequently used for the construction of sequencing libraries for microbial community analysis. This involved targeting the bacterial 16S rRNA gene and fungal ITS2 region to assess the composition and diversity of the microbial communities present in the Huangjiu samples.

#### 2.5.1. 16S/ITS2 Amplicon Analysis and Metagenomic Sequencing

Protocols for amplifying bacterial 16S rRNA genes and fungal internal transcribed spacer (ITS) regions were adapted from [8], with modifications tailored for compatibility with the Huangjiu sample matrix and the study’s research objectives. These regions were selected for their ability to capture the diversity of both bacterial and fungal communities present in the samples.

#### 2.5.2. 16S rRNA Gene Amplification

The V3–V4 hypervariable region of the bacterial 16S rRNA gene was amplified using the 341F (5′-CCTAYGGGRBGCASCAG-3′) and 806R (5′-GACTACNNGGGTATCTAAT-3′) primers, following established protocols [8]. PCR reactions were run in triplicate with an initial denaturation at 95 °C for 3 min, followed by 30 cycles of 95 °C for 30 s, 55 °C for 30 s, and 72 °C for 45 s, concluding with a final extension at 72 °C for 5 min.

#### 2.5.3. ITS2 Region Amplification

For fungal community analysis, the ITS2 region was amplified using primers ITS3 (5′-GCATCGATGAAGAACGCAGC-3′) and ITS4 (5′-CCTCCGCTTATTGATATGC-3′). PCR amplification was performed with an initial denaturation at 98 °C for 2 min, followed by 35 cycles of 98 °C for 10 s, 55 °C for 30 s, and 72 °C for 30 s, with a final extension at 72 °C for 5 min.

#### 2.5.4. Metagenomic Sequencing

Following PCR amplification, amplicons were purified using the AMPure XP system (Shanghai Rui An Biotechnology Co., Ltd., China) to remove any residual primers, dNTPs, and contaminants. The purified amplicons were quantified using a Qubit 2.0 Fluorometer (Thermo Fisher Technology (Shanghai) Co., Ltd., China) and pooled in equimolar concentrations to construct sequencing libraries. The libraries were then sequenced on an Illumina MiSeq platform (Illumina, San Diego, CA, USA) using a 2 × 250 bp paired-end protocol to generate high-quality reads for subsequent bioinformatic analysis.

#### 2.5.5. Bioinformatic Analysis

Raw sequencing reads were processed using the QIIME2 pipeline (version 2020.6), which included quality filtering, chimera removal, and sequence alignment. High-quality sequences were then clustered into operational taxonomic units (OTUs) at a 97% similarity threshold using the SILVA (bacterial 16S) and UNITE (fungal ITS) databases. Taxonomic classification was performed using the naive Bayesian classifier implemented in QIIME2, and alpha diversity analysis was conducted to assess microbial community structure and diversity across samples.

### 2.6. Annotation of Metabolic Pathways

Metabolic pathways were identified by referencing the Kyoto Encyclopedia of Genes and Genomes (KEGG) database. Sequences were initially aligned through the KEGG Automatic Annotation Server (KAAS), after which gene abundance was normalized across samples to facilitate cross-sample comparisons. A gene was classified as significantly enriched when its relative abundance in a sample was at least tenfold higher than in others. These enriched genes were then linked to their respective metabolic pathways in the KEGG database, enabling the identification of distinct metabolic activities in Huangjiu samples brewed with glutinous rice from various regions.

### 2.7. Evaluation of Flavor Using Electronic Sensory (E-Sense) Systems

The flavor profiles of the Huangjiu samples were assessed using an electronic tongue (E-tongue) and an electronic nose (E-nose), both of which provide objective, quantitative measurements of taste and aroma characteristics. These sensory systems have been widely applied in food chemistry for accurate flavor analysis in complex food matrices.

#### 2.7.1. E-Tongue Analysis

Taste characteristics of the Huangjiu samples were measured using the TS-5000Z E-tougue system, equipped with a series of sensors that mimic the human gustatory system. The system includes AAE (sensitivity to umami), CA0 (sensitivity to sourness), AN0 (sensitivity to bitterness), AE1 (sensitivity to acerbity), GL1 (sensitivity to sweetness), and CTO (sensitivity to saltiness compounds) sensors. For each sample, 10 mL of Huangjiu was analyzed at room temperature (25 °C). The system was calibrated using standard solutions prior to sample measurement to ensure reproducibility and reliability of the data.

#### 2.7.2. E-Nose Analysis

The aroma characteristics of the Huangjiu samples were measured using a Surface Acoustic Wave (SAW)-based E-nose (zNose, Electronic Sensor Technology, Thousand Oaks, CA, USA), which detects volatile organic compounds (VOCs) through gas-phase analysis. Each sample was analyzed for 90 s to allow for sufficient detection of volatiles. Data were collected in triplicate and analyzed using gas chromatography principles embedded within the system to generate a detailed VOC profile for each sample.

### 2.8. Identification of Organic Acid and Amino Acid Metabolites

The detection of organic acids and amino acids in the Huangjiu samples was carried out using HPLC and an automatic amino acid analyzer. The employed detection methods of organic acids and amino acids were based on GB 5009.268-2016 [12] and GB 5009.124-2016 [13], respectively.

#### 2.8.1. Organic Acid Identification

The analysis of organic acids was conducted using a high-performance liquid chromatography (HPLC) system (LC-2010, Shimadzu, Kyoto, Japan) equipped with a photodiode array (PDA) detector, set to monitor at 210 nm. The separation of organic acids was performed on a C18 reverse-phase column (4.6 mm × 250 mm, 5 μm), selected for its efficiency in resolving low-molecular-weight organic acids. The mobile phase consisted of 0.01 M sulfuric acid in water, ensuring optimal separation conditions. The flow rate was maintained at 0.6 mL/min, and the column temperature was stabilized at 30 °C to ensure consistent sample processing.

Prior to injection, Huangjiu samples were filtered through 0.45 μm syringe filters to remove particulates. A 20 μL injection volume was used for each sample, with all analyses performed in triplicate. Organic acids were quantified using an external standard method, comparing retention times and peak areas against calibration curves generated from reference standards, including lactic, acetic, citric, and succinic acids. The results were expressed in mg/mL. Standard solutions were prepared by accurately weighing specific amounts of ten organic acids and dissolving them in 100 mL volumetric flasks to achieve the following concentration gradients for both single and mixed standard solutions: Oxalic acid: 0.1 mg/mL, 0.3 mg/mL, 0.6 mg/mL, 1.0 mg/mL, 1.5 mg/mL; tartaric acid: 0.2 mg/mL, 0.6 mg/mL, 1.2 mg/mL, 2.0 mg/mL, 3.0 mg/mL; succinic acid, lactic acid, and acetic acid: 2.0 mg/mL, 3.0 mg/mL, 4.0 mg/mL, 5.0 mg/mL, 6.0 mg/mL; citric acid, malic acid, and acetic acid: 0.5 mg/mL, 1.0 mg/mL, 1.5 mg/mL, 2.0 mg/mL, 3.0 mg/mL; fumaric acid and pyruvic acid: 0.01 mg/mL, 0.05 mg/mL, 0.1 mg/mL, 0.15 mg/mL, 0.2 mg/mL.

#### 2.8.2. Amino Acid Identification

For the analysis of amino acids, an automatic amino acid analyzer (Biochrom 30+, Biochrom, Cambridge, UK) was employed. The instrument utilizes ion-exchange chromatography with post-column ninhydrin derivatization, a standard method for accurate and sensitive amino acid quantification. The Huangjiu samples were hydrolyzed using 6 N hydrochloric acid (HCl) at 110 °C for 24 h to break down protein-bound amino acids into their free forms. After hydrolysis, samples were neutralized and diluted appropriately before injection. The column used for separation was a cation-exchange resin, with the mobile phase composed of sodium citrate buffers of varying pH levels to elute different amino acids sequentially. For each analysis, 100 μL of the sample was injected, and amino acids were detected based on their reaction with ninhydrin, which forms a chromophore detected at 570 nm for primary amino acids and 440 nm for secondary amino acids. Amino acid standards (including alanine, glutamic acid, and leucine) were used to calibrate the system and quantify the amino acids present in the Huangjiu samples, with results expressed in mg/L.

### 2.9. Statistical Analysis

All statistical analyses were performed using R software (version 4.4.1, https://www.r-project.org/, accessed on 15 July 2024), a widely utilized platform in scientific research for statistical computing and data visualization. Various statistical techniques were employed to assess differences in microbial community structures, metabolic profiles, and flavor attributes of Huangjiu samples produced with glutinous rice from diverse regions.

## 3. Results and Discussion

### 3.1. Changes in Physicochemical Indexes in Huangjiu Samples

**Figure 1:**
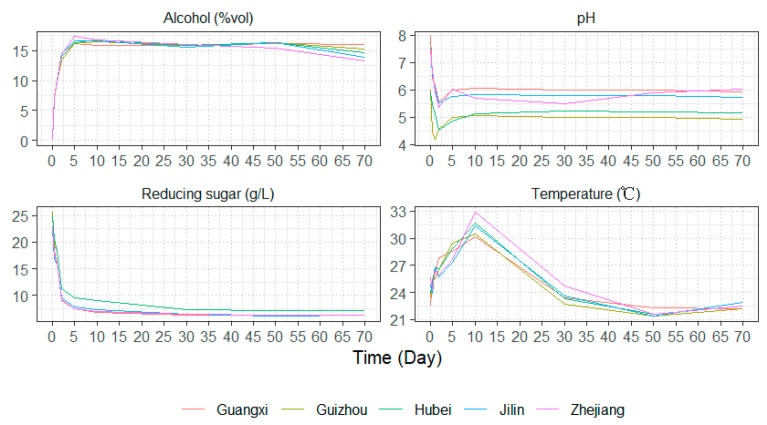
Dynamic changes in all physicochemical indexes during the fermentation of Huangjiu samples, including alcohol content, PH value, reducing sugar content, and temperature.

The dynamic changes in all fermentation parameters are presented in Figure 1. During the early fermentation phase, ethanol and reducing sugar levels exhibited rapid fluctuations—ethanol content increased sharply, while reducing sugar content decreased significantly, both eventually stabilizing in the later stages of fermentation. PH changes followed a distinct trend, dropping rapidly within the first two days, showing a slight increase from days 2 to 5, and then stabilizing. Notably, Hubei and Guizhou Huangjiu maintained consistently lower pH levels throughout the fermentation process compared to the other samples. Temperature dynamics also exhibited a characteristic pattern: it increased rapidly during the first 10 days, followed by a sharp decline below the initial fermentation temperature, only beginning to stabilize around day 30. The rapid changes in reducing sugar and ethanol levels during early fermentation can be attributed to starch gelatinization caused by soaking and heating treatments. This process enhances enzymatic hydrolysis, making starch more accessible for conversion into fermentable sugars. *Aspergillus* molds in wheat Qu, along with added saccharifying enzymes, facilitated starch saccharification, leading to an initial surge in reducing sugars. At the same time, the high availability of nutrients and oxygen—due to stirring and aeration after inoculation—promoted rapid yeast metabolism, resulting in fast ethanol accumulation [14]. However, in the later stages of fermentation, the depletion of reducing sugars limited yeast metabolism, leading to a plateau in ethanol production. However, in the later stages of fermentation, the depletion of reducing sugars limited yeast metabolism, leading to a plateau in ethanol production. The rapid pH drop within the first two days was primarily driven by the active proliferation and metabolism of acid-producing bacteria. The subsequent minor pH rebound between days 2 and 5 may be attributed to autolysis of acidogenic bacteria as ethanol levels peaked, or to temporary nutrient limitations (e.g., reducing sugar depletion), affecting microbial acid production. In the later fermentation stages, organic acids (e.g., lactic acid, acetic acid) reacted with ethanol via esterification, forming flavor compounds such as ethyl lactate and ethyl acetate, which are essential to Huangjiu’s sensory profile. This balance between organic acid generation and esterification contributed to pH stabilization in the final phase of fermentation [15]. The initial temperature rise was linked to the rapid microbial proliferation and active sugar metabolism, particularly by yeasts and saccharifying microorganisms. However, as fermentation progressed, the temperature gradually declined, favoring the formation of esters, which play a crucial role in shaping Huangjiu’s complex aroma and flavor profile.

### 3.2. Microbial Diversity in Huangjiu Samples

**Figure 2:**
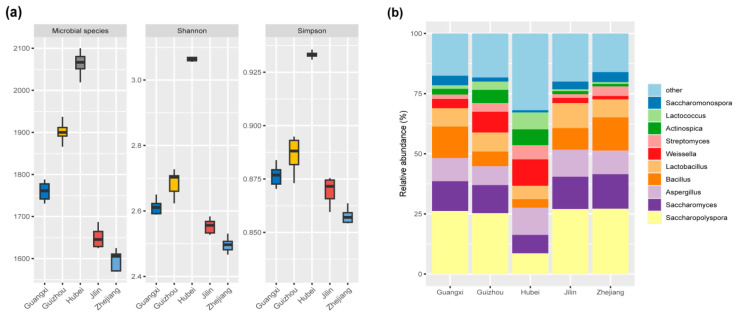
Boxplots of α-diversity indices (**a**), including the Microbial species, Shannon Index, and Simpson Index, illustrating the microbial diversity in Huangjiu samples brewed with different glutinous rice varieties. (**b**) Relative abundance and diversity of microbial genera in Huangjiu samples.

As shown in Figure 2a, the Shannon and Simpson indices, which represent microbial diversity, as well as the number of microbial species (indicative of richness), were highest in Hubei Huangjiu (*p* < 0.05). This suggests that the Hubei samples harbor the most diverse and rich microbial community, followed by Guizhou, Guangxi, Jilin, and Zhejiang Huangjiu. These findings highlight the influence of regional glutinous rice varieties on microbial ecology during fermentation.

Across all Huangjiu samples, 414 microbial genera from 19 phyla were identified, with 10 dominant genera having relative abundances exceeding 2.5% (Figure 2b). The dominant genera included *Saccharopolyspora* (22.90%), *Saccharomyces* (11.88%), *Aspergillus* (9.99%), *Bacillus* (9.08%), *Lactobacillus* (7.81%), *Weissella* (5.40%), and so on. These genera displayed significant variation in abundance across the Huangjiu samples (*p* < 0.05), which suggests that microbial composition is highly dependent on the specific glutinous rice varieties used. Interestingly, *Lactococcus*, *Actinospica*, *Streptomyces*, and *Weissella* are more abundant in Guizhou and Hubei Huangjiu than in Guangxi, Jilin, and Zhejiang Huangjiu. Conversely, *Saccharopolyspora*, *Saccharomyces*, *Aspergillus*, *Bacillus*, *Lactobacillus*, and *Saccharomonospora* are more abundant in Guangxi, Jilin, and Zhejiang Huangjiu than in Guangxi, Jilin, and Zhejiang Huangjiu. The differential abundance of these microbial taxa underscores the regional specificity of microbial communities in Huangjiu fermentation.

*Saccharopolyspora*, an actinomycete, is known for its production of secondary metabolites, including macrolides, quinones, and oligosaccharides, which possess potent insecticidal, antimicrobial, and anticancer activities [16,17]. The abundance of *Saccharopolyspora* in Zhejiang, Guangxi, and Jilin samples suggests a potential medical and bioactive benefit associated with these regional Huangjiu products. Moreover, *Saccharopolyspora* plays a vital role in wheat qu and Huangjiu fermentation, aiding in the breakdown of cellulose and starch [18,19].

The fungal genus *Aspergillus* is crucial for the saccharification process, producing enzymes that convert glutinous rice starch into fermentable sugars, which are later metabolized by *Saccharomyces* for alcohol and ester production, key components in shaping Huangjiu’s flavor [2,20,21]. *Aspergillus* molds also play a crucial role in the fermentation of traditional fermented foods, such as soy sauce. Unlike Huangjiu fermentation, soy sauce production requires high salt concentrations, meaning the *Aspergillus* strains used must exhibit enhanced salt tolerance and the ability to produce acid-resistant proteases to facilitate protein breakdown under saline conditions [22]. Huangjiu fermentation primarily relies on *Saccharomyces cerevisiae*, whereas other fermented beverages utilize diverse non-Saccharomyces yeast strains to modulate their sensory characteristics. For example, Nuno et al. explored the use of various non-Saccharomyces yeast strains in beer brewing, demonstrating their distinct contributions to flavor complexity and aroma profiles [23].

LAB, such as *Lactobacillus* and *Weissella*, significantly influence the fermentation process by producing organic acids like lactic, acetic, and succinic acids, which act as flavor precursors [24]. Additionally, LABs enhance food safety by generating antimicrobial compounds that inhibit spoilage and pathogenic microbes [25]. The regional differences in LAB abundance across the Huangjiu samples could explain the variations in organic acid content, which directly influence the sourness and acidity of the final product. Huangjiu samples from Guizhou and Hubei had higher LAB levels, which likely contributed to their distinct acidic flavor profiles. In beer fermentation, lactic acid bacteria (LAB) can be introduced to produce sour beers. However, an ongoing challenge remains in selecting LAB strains with high activity and stability that can effectively function under the conditions of beer brewing [26,27]. In wine fermentation, LAB play a crucial role in malolactic fermentation (MLF), where malic acid is converted into lactic acid, resulting in a smoother acidity profile and improved flavor balance [28].

Influenced by glutinous rice varieties and regional environmental factors, Huangjiu has a unique microbial succession pattern during fermentation, which is different from other fermented beverages. Baijiu fermentation, despite being a Chinese alcoholic beverage, involves an open fermentation process dominated by complex filamentous fungi (e.g., *Rhizopus*) and a broader range of LAB, which differs significantly from the controlled fermentation of Huangjiu [29]. Makgeolli (Korean rice wine) shares similarities in microbial diversity, but its fermentation is typically more influenced by *Pediococcus* and *Leuconostoc*, which were less prevalent in our Huangjiu samples [30]. Sake fermentation, which also utilizes rice as a substrate, is dominated by *Saccharomyces cerevisiae* and *Aspergillus oryzae*, but lacks the significant presence of lactic acid bacteria (LAB) that we observed in Huangjiu [31].

### 3.3. Metabolic Pathway of Huangjiu Samples

**Figure 3:**
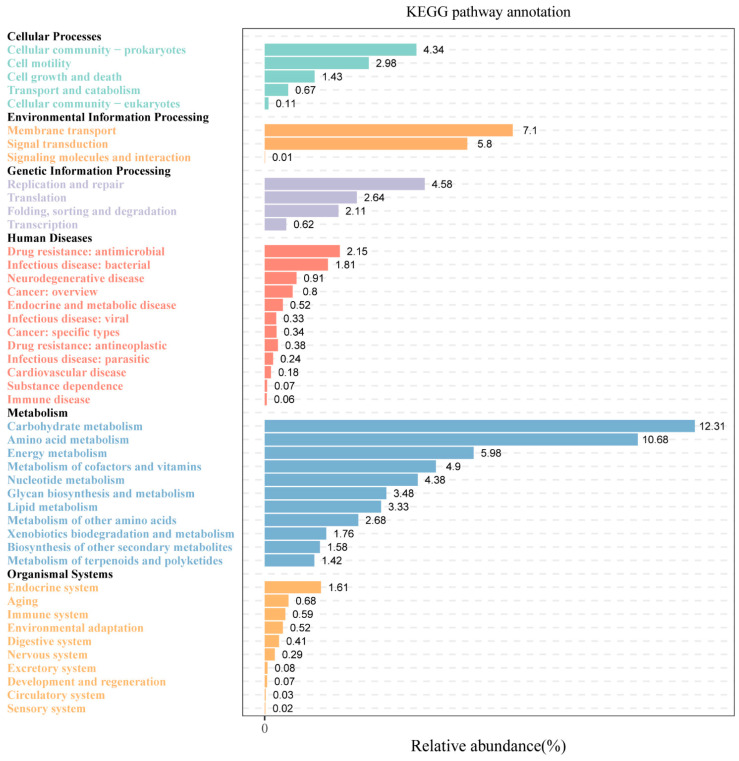
Overview of the metabolic pathways identified in all Huangjiu samples using KEGG pathway annotation, illustrating the functional categories and dominant metabolic pathways.

To elucidate the metabolic traits of microorganisms participating in Huangjiu fermentation, we conducted an analysis of microbial community metabolic pathways across all samples. Through KEGG annotation, 45 distinct metabolic pathways were identified and grouped into six primary functional categories (Figure 3). The largest category was related to general metabolism, highlighting the robust metabolic activity present within the microbial populations in Huangjiu. Among these, five key metabolic pathways, each with an average relative abundance exceeding 5%, were identified: carbohydrate metabolism, amino acid metabolism, membrane transport, energy metabolism, and signal transduction.

The Wilcoxon test revealed significant differences in the abundance of these dominant pathways among the five regions (Table 3). Carbohydrate and amino acid metabolism were more abundant in Guizhou and Hubei Huangjiu, compared to samples from Zhejiang, Guangxi, and Jilin. Conversely, signal transduction and energy metabolism were more prevalent in Zhejiang, Guangxi, and Jilin, suggesting that different microbial activities are influenced by the regional variations in glutinous rice. The richness of metabolic pathways involved in carbohydrate and amino acid metabolism in Guizhou and Hubei Huangjiu could be attributed to the composition of grains. Grains provide essential nutrients, such as starch and protein, which are degraded by microbial enzymes during fermentation, directly affecting the growth and metabolism of the microbial community [32,33]. Glutinous rice, a key grain raw material in Huangjiu, is rich in amylopectin (over 98%), making it highly suitable for fermentation due to its ease of hydrolysis and ability to provide energy substrates for microbial metabolism [34,35,36,37]. In this study, we also used the saccharifying enzyme, which is also known as glucose amylase. This enzyme hydrolyzes the α-1,4 and α-1,6 glycosidic bonds at the non-reducing ends of starch molecules, converting them into glucose. Its use not only reduces the amount of wheat koji required but also prevents overactive yeast fermentation that can lead to premature yeast senescence [38].

In this study, Guizhou and Hubei Huangjiu demonstrated a higher abundance of LAB and carbohydrate metabolism pathways than other Huangjiu samples. We hypothesize that the glutinous rice from these regions contains higher levels of amylopectin, which enhances carbohydrate metabolism, thereby promoting the growth of LAB. This is supported by findings from KEGG pathway analysis, which indicate that LAB possesses specialized pathways for starch metabolism and can secrete extracellular enzymes to hydrolyze starch [39]. In addition, two lactate dehydrogenases involved in carbohydrate metabolism were identified in this study, which are glycolytic enzymes responsible for converting pyruvate into D-lactic acid and L-lactic acid. These findings indicate a strong carbohydrate metabolism in LAB, which is consistent with the higher abundance of LAB in Guizhou and Hubei samples. Throughout the starch saccharification, amylopectin is quickly broken down into glucose, fructose, maltose, and other reducing sugars, which serve as vital energy sources for LAB. Then, LAB metabolizes these sugars through fermentation, producing lactic, acetic, and citric acids, which are responsible for the decreased PH values observed in Huangjiu from Guizhou and Hubei regions. Glutinous rice from other regions may contain a higher proportion of amylose, which is more resistant to enzymatic hydrolysis. This results in a lower availability of reducing sugars, thereby limiting the carbon source supply for LAB metabolism. Consequently, we observed that Huangjiu brewed with these rice varieties exhibited lower LAB abundance and higher PH values, indicating that starch composition significantly influences microbial dynamics and acid production during fermentation.

Another factor contributing to the enriched carbohydrate metabolism in Guizhou and Hubei Huangjiu is the presence of *Aspergillus*, a key fungus involved in the saccharification of grains during the early stages of Huangjiu fermentation. *Aspergillus* secretes amylase, which hydrolyzes starch into fermentable sugars, providing substrates for the metabolic activities of other microorganisms [40]. Additionally, *Aspergillus* secretes protease, which breaks down proteins in glutinous rice into amino acids and oligopeptides, further supporting the growth of other microorganisms and contributing to the high amino acid metabolism observed in Guizhou and Hubei samples [40]. The interplay between *Aspergillus* and LAB appears to be a key factor in the microbial dynamics of Huangjiu fermentation. As *Aspergillus* hydrolyzes starch and proteins, LAB utilizes the resulting sugars and amino acids to generate metabolic byproducts, such as organic acids, which influence the sensory characteristics of Huangjiu. This synergistic relationship likely explains the enhanced carbohydrate and amino acid metabolism in Guizhou and Hubei samples.

### 3.4. Flavor Characteristics of Huangjiu Samples Based on E-Nose Analysis

Sensory analysis is a routine quality assessment conducted before Huangjiu is placed on the market. It is widely used in the food industry to ensure product consistency, prevent market rejection, and guide adjustments in raw material selection or process optimization. This analysis helps manufacturers maintain flavor stability and consumer acceptability over different production batches. In our study, we employed electronic sensory techniques (E-nose and E-tongue) to evaluate the aroma and taste profiles of Huangjiu, providing an objective and reproducible approach to assess flavor differences across samples. This methodological approach aligns with routine industry practices, ensuring that the results are relevant to both scientific research and industrial applications [9].

**Figure 4:**
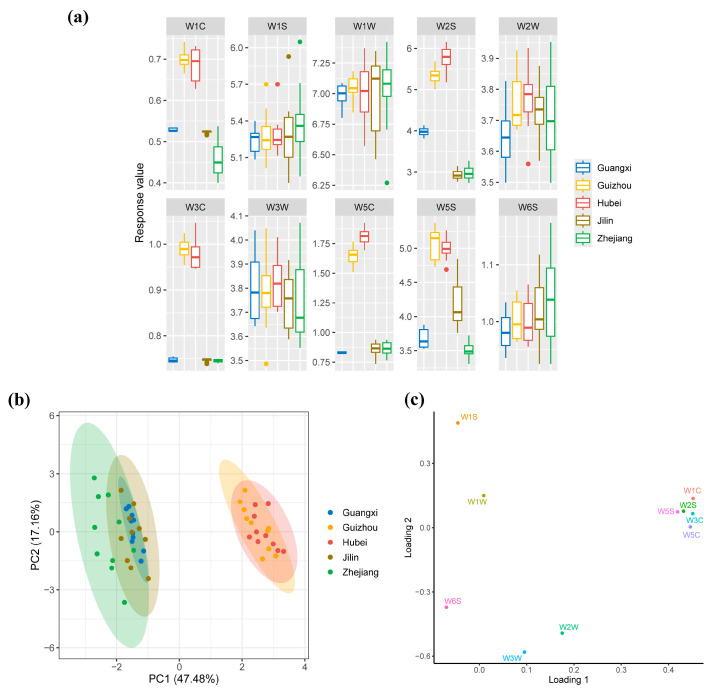
(**a**) Box plot for aroma profiles of Huangjiu prepared using different glutinous rice varieties. (**b**) PCA score plot based on the aroma profiles, separating the Huangjiu samples according to their regional glutinous rice varieties. (**c**) PCA loading plot of aroma profiles, visualizing the contributions of different volatile compounds to the overall aroma of the Huangjiu samples.

As depicted in Figure 4, the response values of five sensors—W1C, W3C, and W5C (detecting aromatic compounds) and W2S and W5S (responding to sulfur-containing organic compounds)—showed significant differences among the five Huangjiu samples (*p* < 0.05). In particular, Hubei and Guizhou Huangjiu exhibited notably higher sensor responses compared to those from Zhejiang, Jilin, and Guangxi (*p* < 0.05), indicating a more complex and intense aroma profile in the former two varieties. Moreover, the sum of the response values for all sensors in Hubei and Guizhou Huangjiu was higher than that in the other samples, indicating a greater abundance of aromatic compounds in these varieties. Generally, these observations suggest that regional differences in glutinous rice varieties significantly affect the volatile composition of Huangjiu, resulting in distinct flavor profiles.

The aroma characteristics of the five Huangjiu varieties were further analyzed using principal component analysis (PCA) to explore patterns and groupings based on sensor responses. As shown in Figure 4b, the PCA score plot reveals two distinct clusters: one consisting of Huangjiu samples from Zhejiang, Jilin, and Guangxi, and the other comprising samples from Guizhou and Hubei. The first cluster is located on the left side of the plot, while the second is positioned on the right. This clear separation indicates significant differences in the aromatic profiles of the two groups of Huangjiu, likely driven by differences in the glutinous rice varieties used for fermentation. Further analysis of the PCA loading plot (Figure 4c) revealed that the aromatic profiles of Zhejiang, Jilin, and Guangxi Huangjiu were more closely associated with the responses of WW sensors and some WS sensors, which are known to detect sulfur-containing organic compounds. Conversely, the aromatic profiles of Guizhou and Hubei Huangjiu were more strongly influenced by WC sensors and additional WS sensors, which are more sensitive to aromatic hydrocarbons and other complex volatile compounds.

The results from the E-nose analysis provide important insights into the aroma differentiation of Huangjiu samples. The higher response values for aromatic and sulfur compounds in Hubei and Guizhou Huangjiu suggest a more diverse and complex volatile profile compared to Zhejiang, Jilin, and Guangxi Huangjiu. This aligns with previous research indicating that a variety of variations in raw materials, such as glutinous rice or sorghum, can significantly impact the production of key volatile compounds during fermentation [41]. The abundance of sulfur-containing compounds, detected by WS sensors, likely contributes to the subtle differences in aroma between the Zhejiang, Jilin, and Guangxi samples, while the more pronounced aromatic compounds detected in Hubei and Guizhou samples can be attributed to the influence of microbial metabolism, particularly the action of *Aspergillus* and *Saccharomyces*, which play a critical role in the production of esters and alcohols during fermentation [42].

### 3.5. Flavor Characteristics of Huangjiu Samples Based on E-Tongue Analysis

**Figure 5:**
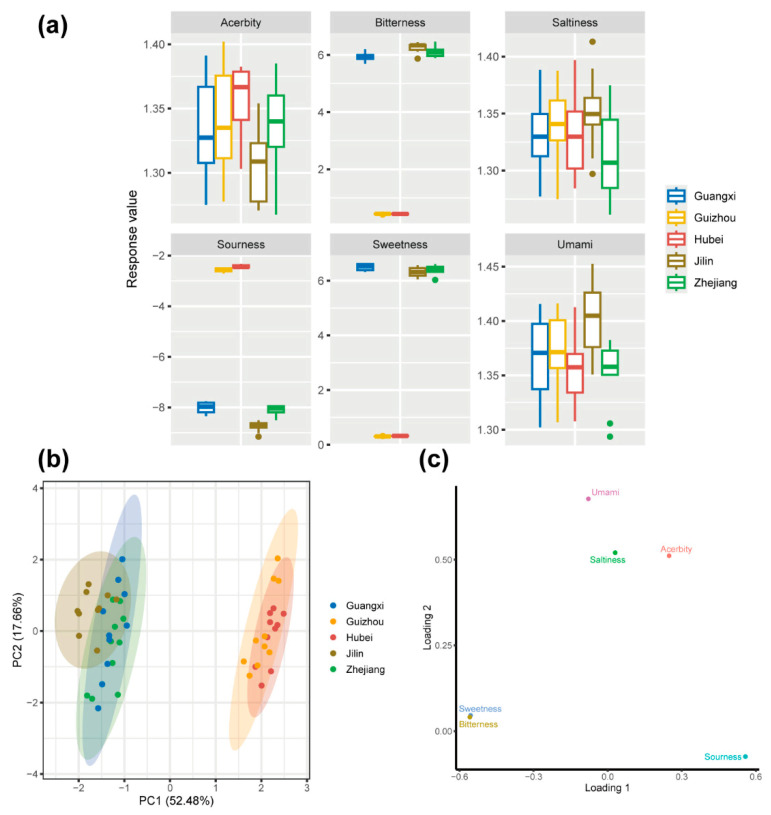
(**a**) Boxplot for taste profiles of Huangjiu prepared using different glutinous rice varieties. (**b**) PCA score plot of taste profiles, illustrating the differentiation of Huangjiu samples based on their sensory characteristics. (**c**) PCA loading plot of taste profiles, showing the contribution of various sensory attributes (e.g., sweetness, bitterness, sourness) to the overall taste profile of the Huangjiu samples.

As shown in Figure 5a, the response values for sweetness, bitterness, and sourness varied significantly across the five Huangjiu samples (*p* < 0.05). Hubei and Guizhou Huangjiu exhibited higher response values for sourness (*p* < 0.05), while Guangxi, Jilin, and Zhejiang Huangjiu displayed higher response values for sweetness and bitterness (*p* < 0.05). These differences suggest that glutinous rice varieties have a significant impact on certain taste attributes, particularly sourness, bitterness, and sweetness, although the effects on umami, acerbity, and saltiness were relatively minor.

The elevated sourness in Hubei and Guizhou Huangjiu may be attributed to the higher abundance of LAB in these samples, as LAB are known to produce organic acids such as lactic and acetic acids during fermentation. The microbial communities in Hubei and Guizhou are enriched in LAB, which likely contributes to the higher levels of organic acids and, consequently, the more pronounced sourness observed in these regions’ Huangjiu. Carbohydrate metabolism plays a critical role in driving the production of these organic acids. In Guizhou and Hubei, the glutinous rice used in Huangjiu fermentation is rich in amylopectin, a readily hydrolyzable starch that provides the sugars needed for microbial growth and acid production. This relationship between amylopectin-rich rice and increased LAB activity could explain the heightened sourness detected by the E-tongue in these samples. On the other hand, Guangxi, Jilin, and Zhejiang Huangjiu had higher response values for sweetness and bitterness. The elevated sweetness is likely due to the presence of residual sugars or sugar alcohols that result from incomplete fermentation or microbial activity that produces compounds like glycerol, which can impart sweetness to fermented beverages [43]. The bitterness in these samples may be attributed to the presence of specific microbial metabolites, including higher alcohols, peptides, and phenolic compounds. Studies have shown that the metabolic activities of yeasts, such as Saccharomyces, contribute to the formation of bitter-tasting higher alcohols during fermentation [44]. Additionally, peptides formed during protein degradation in the fermentation process can enhance bitterness [45].

PCA was performed on the Huangjiu samples based on E-nose data (Figure 5b). As shown in Figure 5b, the group of Guizhou and Hubei Huangjiu and the group of other Huangjiu showed an obvious horizontal separation trend. The horizontal separation observed in the PCA plot suggests that glutinous rice varieties play a significant role in influencing the taste profiles of Huangjiu. Combined with the PCA score plot (Figure 5b) and PCA loading plot (Figure 5c), we found that Guizhou and Hubei Huangjiu were distributed on the left side of the plot and characterized by sourness, while Guangxi, Jilin, and Zhejiang Huangjiu were distributed on the right side of the plot and characterized by bitterness and sweetness.

### 3.6. Organic Acid and Amino Acid Metabolites in Huangjiu Samples

Organic acids and amino acids are essential contributors to the sensory profile of Huangjiu. Organic acids, primarily responsible for sourness, also play a role in inhibiting bacterial contamination during fermentation, ensuring the quality and stability of the wine. Furthermore, these organic acids, when reacting with alcohols, lead to ester formation, enriching the complexity of Huangjiu’s flavor profile [24,46]. Amino acids not only support microbial growth but also serve as key precursors to various flavor compounds, influencing taste characteristics like sweetness, bitterness, and umami [46]. Considering their significance, a thorough qualitative and quantitative analysis of both organic and amino acids is essential for fully understanding the flavor dynamics of Huangjiu.

A total of 19 amino acids were identified in the Huangjiu samples (Table 4), classified into sweet, umami, bitter, and astringent categories. The overall amino acid concentration was significantly greater (*p* < 0.05) in the Zhejiang, Guangxi, and Jilin samples compared to those from Guizhou and Hubei. Among the detected amino acids, glycine, alanine, proline, glutamic acid, arginine, leucine, and tyrosine were the most prevalent. Nine organic acids were identified across the Huangjiu samples (Table 5). Guizhou and Hubei Huangjiu contained significantly higher levels of total organic acids (*p* < 0.05) compared to Zhejiang, Guangxi, and Jilin. The most abundant organic acids were lactic acid (47.2%), acetic acid (20.3%), and citric acid (15.5%).

**Figure 6:**
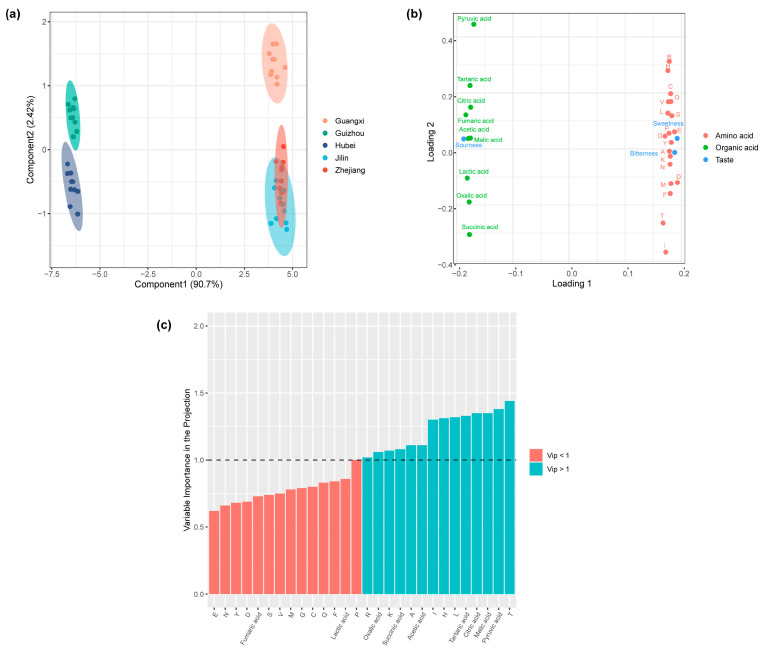
(**a**) Partial least squares (PLS) score plot visualizing the difference among Huangjiu samples. (**b**) PLS loading plot showing the contributions of specific metabolites to the taste profiles of Huangjiu. (**c**) Identification of key organic acid and amino acid metabolites (VIP > 1) through Variable Importance in Projection (VIP) analysis, indicating their significant influence on taste.

To explore the correlation between amino acids, organic acids, and taste characteristics, a partial least squares (PLS) regression analysis was conducted. In this analysis, organic acids and amino acids were treated as predictors (X-block), while the dominant taste indicators (sweetness, bitterness, and sourness) served as response variables (Y-block) (Figure 6a–c). The model performed well, with R^2^x, R^2^y, and Q^2^ values of 0.96, 0.91, and 0.89, respectively, indicating a strong predictive capability. The score plot shows that the Huangjiu samples show an obvious separation trend in the horizontal direction (Figure 6a). Combined with the loading plot (Figure 6b), we find that Guizhou and Hubei Huangjiu clustered on the left, characterized by higher concentrations of organic acids and a dominant sour taste. In contrast, Zhejiang, Guangxi, and Jilin Huangjiu grouped on the right, associated with higher concentrations of amino acids and more pronounced sweetness and bitterness. To determine which metabolites contributed most to the flavor profile, variable importance in projection (VIP) analysis was performed (Figure 6c). Out of the 19 detected metabolites, 7 amino acids, and 7 organic acids showed the highest contribution to taste characteristics (VIP > 1). These dominant metabolites include alanine, threonine, histidine, isoleucine, leucine, lysine, pyruvate, oxalic acid, succinic acid, malic acid, tartaric acid, citric acid, and acetic acid. The identification of these key metabolites highlights their critical role in defining the flavor complexity of Huangjiu.

### 3.7. Correlation Analysis of Dominant Amino Acid/Organic Acid Metabolites and Dominant Microbial Genera in Huangjiu Samples

**Figure 7:**
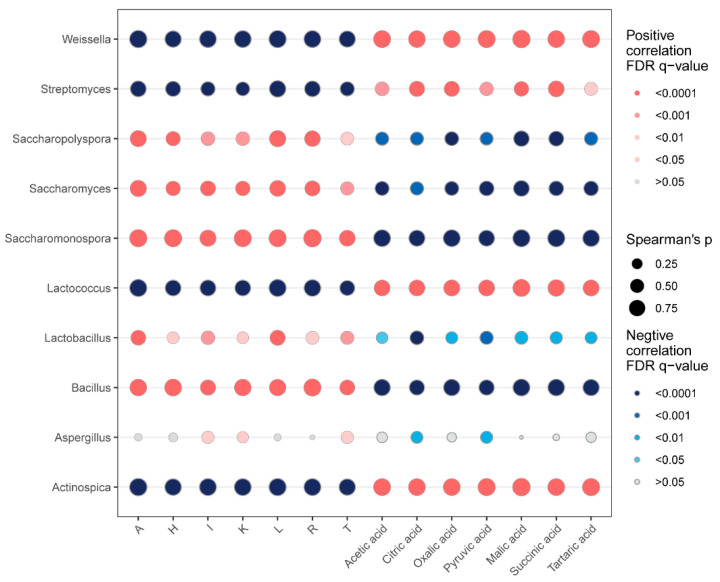
Spearman’s rank correlation analysis, illustrating the relationships between dominant microbial genera and dominant organic acid/amino acid metabolites in Huangjiu samples.

To investigate the relationship between dominant microbial genera and key amino acid and organic acid metabolites in Huangjiu, Spearman’s rank correlation analysis was performed (Figure 7). This analysis provides insights into how microbial community dynamics influence the flavor profile of Huangjiu by impacting the concentrations of essential metabolites.

The genera *Weissella*, *Streptomyces*, *Lactococcus*, and *Actinospica* were found to be positively correlated with organic acid abundance (*p* < 0.05) and negatively correlated with amino acid concentrations (*p* < 0.05). Both *Weissella* and *Lactococcus* are members of the LAB group, which play a crucial role in the fermentation process by producing organic acids. The high abundance of organic acids in LAB-rich Huangjiu samples, particularly Guizhou and Hubei Huangjiu, is likely a direct result of LAB metabolic activity. LAB rely on carbohydrates as their primary substrate, converting them into organic acids through various carbohydrate metabolic pathways. In homolactic fermentation, glucose is converted into pyruvate through the EMP pathway, followed by its reduction to lactic acid, catalyzed by lactate dehydrogenase [47]. In contrast, in heterolactic fermentation, pyruvate produced by the EMP pathway is further metabolized into ethanol and acetic acid, facilitated by pyruvate formate lyase and pyruvate oxidase [48]. Additionally, LAB can consume amino acids as nutrients, thereby reducing the concentration of amino acids in wine [49]. The consumption of essential amino acids such as lysine, arginine, and glutamate by LAB during fermentation limits the accumulation of these amino acids, which can explain the negative correlation between LAB and amino acid concentration [50]. This suggests that LAB not only modulate the sourness of Huangjiu by producing organic acids but also affect the overall amino acid content through their metabolic processes.

Conversely, the presence of *Saccharopolyspora*, *Saccharomyces*, *Aspergillus*, *Bacillus*, *Lactobacillus*, and *Saccharomonospora* was positively correlated with amino acid concentrations (*p* < 0.05) and negatively correlated with organic acid levels (*p* < 0.05). This correlation suggests that these microorganisms, particularly *Saccharopolyspora*, *Saccharomonospora*, *Saccharomyces*, and *Bacillus*, contribute more significantly to amino acid production during fermentation. *Saccharopolyspora* and *Saccharomonospora*, two genera of actinomycetes, are known for secreting proteases that degrade proteins into amino acids, and these amino acids not only support the growth of other microorganisms but also contribute to the overall flavor profile of Huangjiu [18,51]. The role of *Saccharopolyspora* and *Saccharomonospora* in enhancing amino acid content aligns with their observed prevalence in Zhejiang, Guangxi, and Jilin Huangjiu, which displayed higher concentrations of amino acids. *Saccharomyces*, a yeast species widely used in alcohol fermentation, is well-known for its ethanol production, but it also contributes to amino acid synthesis. Ethanol itself acts as a precursor for the formation of several amino acids. Additionally, *Saccharomyces* undergoes autolysis, releasing proteins, peptides, and amino acids into the extracellular matrix during fermentation, thus enriching the amino acid content of the wine [49]. *Bacillus* can enhance the population and viability of LAB by releasing proteases and other bioactive extracellular compounds. These enzymes facilitate the breakdown of proteins into peptides and amino acids, providing critical nutrients that support the growth and metabolic activity of LAB [52].

Overall, this correlation analysis highlights the critical role of microbial community structure in influencing the metabolite profile and, consequently, the flavor of Huangjiu. The presence of LAB genera is strongly associated with higher organic acid concentrations, contributing to a pronounced sour taste, particularly in Guizhou and Hubei Huangjiu. In contrast, *Saccharopolyspora*, *Saccharomonospora*, *Saccharomyces*, and *Bacillus* are linked to higher amino acid concentrations, leading to enhanced sweetness and bitterness, as observed in Zhejiang, Guangxi, and Jilin Huangjiu. In our study, lactic acid bacteria, including *Weissella* and *Lactococcus*, utilize amino acids as nutrients but lack the enzymes required for de novo synthesis of amino acids. As a result, they rely on amino acids degraded from the raw materials, generating various organic acids that contribute to the sour taste of Huangjiu. Furthermore, the grains used in Huangjiu fermentation typically have a high starch content but low protein and fat levels, leading to a lower amino acid content in the final product and limiting the nutritional value of the wine. This phenomenon has also been confirmed in other fermented beverages. For example, in Baijiu, *Lactococcus* and *Pediococcus* are the primary acid-producing bacteria, capable of rapidly producing acids by utilizing amino acids degraded from the raw materials [29]. Additionally, Zhou et al. demonstrated that the addition of exogenous proteins to the millet Huangjiu fermentation environment increased the supply of amino acids, balancing the significant consumption of amino acids by lactic acid bacteria and enhancing the amino acid content in the Huangjiu product. This approach provides valuable insights into supporting microorganisms with the necessary nutrients while preserving the high nutritional value of fermented beverages [53].

## 4. Conclusions

This study provides a comprehensive investigation into the effects of glutinous rice varieties from different regions on the microbial community structure, metabolic pathways, and flavor characteristics of Huangjiu through metagenomic analysis, microbial diversity assessment, and metabolite profiling. A total of 10 dominant microbial genera were identified across the Huangjiu samples, with the microbial diversity and richness being significantly higher in Guizhou and Hubei Huangjiu compared to Zhejiang, Guangxi, and Jilin Huangjiu. The abundance of LAB in Hubei and Guizhou samples was strongly correlated with increased levels of organic acids, which imparted a more pronounced sour taste. Conversely, the elevated amino acid content in Zhejiang, Guangxi, and Jilin Huangjiu contributed to a sweeter and more bitter taste profile.

These findings highlight the significant impact of glutinous rice varieties on microbial metabolism and flavor development in Huangjiu, providing new insights into how raw material selection can be strategically utilized to optimize the sensory attributes of this traditional Chinese rice wine. For example, if manufacturers aim to produce Huangjiu with a distinctive acidic profile, they could develop a tailored fermentation starter enriched with high organic acid-producing LAB strains isolated from Huangjiu brewed with Guizhou or Hubei glutinous rice. This targeted approach would enable controlled acidification, enhancing the desired sourness and flavor complexity of the final product. Additionally, these findings can be applied in large-scale production for quality control. For example, standardizing the selection of raw materials (glutinous rice varieties) based on their influence on microbial metabolism can help improve flavor consistency across production batches. At the same time, implementing real-time microbial and metabolite monitoring techniques (e.g., metagenomics-based microbial tracking or metabolomics-guided fermentation adjustments) could provide data-driven fermentation control, ensuring optimal product quality. However, the mechanistic link between microbial activity and flavor compound production remains to be fully explored. While we have established correlations between certain microbial genera and organic/amino acids, the precise metabolic pathways and enzymes responsible for these transformations are not yet fully understood. To address this gap, future research could benefit from functional metagenomic analysis or transcriptomic profiling. These approaches would allow for a direct assessment of microbial metabolic contributions, providing insights into the specific genes and pathways involved in flavor compound production, particularly those related to organic acid metabolism, amino acid conversion, and ester formation. We believe that integrating multi-omics techniques, such as metagenomics, transcriptomics, and metabolomics, would significantly enhance our understanding of microbial-driven flavor formation in Huangjiu fermentation.

## Figures and Tables

**Table 1:** Conditions of vegetative stage of glutinous rice in different regions.

Glutinous Rice Variety	Region	Climate Type	Varietal Type	Sowing Period	Growth Duration (Days)
Shaonuo 9714	Shaoxing, Hejiang	Subtropical Monsoon Climate	Japonica Glutinous	May	147.7
Enuo No. 9	Wuhan, Hubei	Subtropical Humid Monsoon Climate	Indica Glutinous	June	125.8
Jizhan No. 3	Changchun, Jilin	Temperate Continental Semi-Humid Monsoon Climate	Japonica Glutinous	April	143
Qiannuo You 11	Guiyang, Guizhou	Subtropical Humid Monsoon Climate	Indica Glutinous	April	152.9
Guangxiang Nuo 2	Nanning, Guangxi	Subtropical Monsoon Climate	Early Indica Glutinous	March	128.5

**Table 2:** The proportion of raw materials in Huangjiu samples.

Raw Material	Weight (g)
Cooked glutinous rice	900
Mixed wheat koji	625
Highly active dry yeast	100
Saccharifying enzyme	0.15
Jianhu water	500

**Table 3:** Comparison of key metabolic pathways between Huangjiu samples brewed from different glutinous rice varieties, highlighting significant metabolic differences.

Pathway ^a^	Group1	Group2	Median1 (%)	Median2 (%)	*p*	Padj ^b^
C	Guangxi	Guizhou	10.07	14.78	0.00031	0.00088
	Guangxi	Hubei	10.07	16.82	0.00010	0.00044
	Guizhou	Hubei	14.78	16.82	0.00855	0.01457
	Guizhou	Jilin	14.78	10.40	0.00031	0.00088
	Guizhou	Zhejiang	14.78	9.81	0.00008	0.00044
	Hubei	Jilin	16.82	10.40	0.00010	0.00044
	Hubei	Zhejiang	16.82	9.81	0.00002	0.00036
A	Guangxi	Guizhou	10.87	12.26	0.02857	0.04314
	Guangxi	Hubei	10.87	11.92	0.00952	0.01611
	Guizhou	Jilin	12.26	11.05	0.00606	0.01082
	Guizhou	Zhejiang	12.26	10.95	0.00404	0.00739
	Hubei	Jilin	11.92	11.05	0.00117	0.00238
	Hubei	Zhejiang	11.92	10.95	0.00067	0.00147
M	Guangxi	Guizhou	7.69	6.87	0.00040	0.00098
	Guangxi	Hubei	7.69	5.54	0.00040	0.00098
	Guizhou	Hubei	6.87	5.54	0.00049	0.00120
	Guizhou	Jilin	6.87	7.92	0.00008	0.00044
	Guizhou	Zhejiang	6.87	7.49	0.00247	0.00473
	Hubei	Jilin	5.54	7.92	0.00008	0.00044
	Hubei	Zhejiang	5.54	7.49	0.00008	0.00044
	Jilin	Zhejiang	7.92	7.49	0.01041	0.01742
E	Guangxi	Guizhou	6.29	4.71	0.00017	0.00059
	Guangxi	Hubei	6.29	4.39	0.00031	0.00088
	Guizhou	Hubei	4.71	4.39	0.00008	0.00044
	Guizhou	Jilin	4.71	6.36	0.00008	0.00044
	Guizhou	Zhejiang	4.71	6.26	0.00008	0.00044
	Hubei	Jilin	4.39	6.36	0.00016	0.00059
	Hubei	Zhejiang	4.39	6.26	0.00016	0.00059
S	Jilin	Zhejiang	6.04	5.75	0.01998	0.03111

a. The functional categories codes of metabolic pathways are as follows: amino acid metabolism (A); carbohydrate metabolism (C); energy metabolism (E); membrane transport (M); S = Signal transduction (S). b. The *p* values were corrected using the Benjamini method.

**Table 4:** Amino acids in Huangjiu samples.

		Concentration (mg/L)				
Amino Acid		Zhejiang	Hubei	Guangxi	Guizhou	Jilin
Sweet	Glycine	247	191	239	194	237
	Alanine	492	378	482	410	499
	Serine	48	36	48	38	47
	Threonine	88	79	89	73	93
	Cystine	14	11	15	11	14
	Methionine	15	12	14	12	15
	Proline	308	229	294	236	289
Umami	Aspartic acid	241	196	237	189	245
	Glutamic acid	437	345	441	349	446
	Asparagine	11	9	11	9	11
	Glutamine	94	71	95	73	90
Bitter	Histidine	89	71	98	69	88
	Arginine	401	315	425	332	400
	Valine	173	142	175	148	171
	Isoleucine	112	94	107	89	118
	Leucine	273	219	279	238	285
	Phenylalanine	248	199	237	202	250
	Lysine	216	177	213	164	209
Acerbity	Tyrosine	199	148	196	148	192
	Sum	3706	2920	3695	2985	3700

**Table 5:** Organic acids in Huangjiu samples.

	Concentration (mg/mL)				
Organic Acid	Zhejiang	Hubei	Guizhou	Guangxi	Jilin
Pyruvic acid	0.06	0.07	0.07	0.05	0.05
Oxalic acid	0.29	0.37	0.36	0.31	0.31
Succinic acid	0.30	0.39	0.41	0.33	0.33
Malic acid	0.26	0.30	0.31	0.25	0.23
Tartaric acid	0.79	0.93	0.90	0.75	0.71
Citric acid	1.57	1.94	1.86	1.47	1.65
Acetic acid	2.03	2.57	2.44	2.06	2.00
Lactic acid	4.77	5.68	5.96	4.66	4.77
Fumaric acid	0.04	0.05	0.05	0.04	0.04
Sum	10.11	12.30	12.36	9.92	10.10

## Data Availability

The original contributions presented in this study are included in the article. Further inquiries can be directed to the corresponding author.

## References

[1] Yang Y., Hu W., Xia Y., Mu Z., Tao L., Song X., Zhang H., Ni B., Ai L. (2020). Flavor formation in Chinese rice wine (Huangjiu): Impacts of the flavor-active microorganisms, raw materials, and fermentation technology. Front. Microbiol..

[2] Chen L., Ren L., Li D., Ma X. (2021). Analysis of microbiomes in three traditional starters and volatile components of the Chinese rice wines. Food Sci. Biotechnol..

[3] Chen C., Liu Y., Tian H., Ai L., Yu H. (2020). Metagenomic analysis reveals the impact of JIUYAO microbial diversity on fermentation and the volatile profile of Shaoxing-jiu. Food Microbiol..

[4] Shen C., Yu Y., Zhang X., Zhang H., Chu M., Yuan B., Guo Y., Li Y., Zhou J., Mao J. (2024). The dynamic of physicochemical properties, volatile compounds and microbial community during the fermentation of Chinese rice wine with diverse cereals. Food Res. Int..

[5] Yu H., Li Q., Guo W., Ai L., Chen C., Tian H. (2023). Unraveling the difference in flavor characteristics of Huangjiu fermented with different rice varieties using dynamic sensory evaluation and comprehensive two-dimensional gas chromatography–quadrupole mass spectrometry. Front. Nutr..

[6] Huang L., Tan H., Zhang C., Li Q., Liu Q. (2021). Starch biosynthesis in cereal endosperms: An updated review over the last decade. Plant Commun..

[7] Yu H., Li Z., Zheng D., Chen C., Ge C., Tian H. (2024). Exploring microbial dynamics and metabolic pathways shaping flavor profiles in Huangjiu through metagenomic analysis. Food Res. Int..

[8] Hong X., Chen J., Liu L., Wu H., Tan H., Xie G., Xu Q., Zou H., Yu W., Wang L. (2016). Metagenomic sequencing reveals the relationship between microbiota composition and quality of Chinese Rice Wine. Sci. Rep..

[9] Cho S., Moazzem M.S. (2022). Recent applications of potentiometric electronic tongue and electronic nose in sensory evaluation. Prev. Nutr. Food Sci..

[10] (2018). Huangjiu.

[11] (2016). Determination of Ethanol Concentration in National Standard Wine for Food Safety.

[12] (2016). Determination of Multi-Element in Food of National Food Safety Standard.

[13] (2016). Determination of Amino Acids in Food of National Food Safety Standard.

[14] Liu S., Yang L., Zhou Y., He S., Li J., Sun H., Yao S., Xu S. (2019). Effect of mixed moulds starters on volatile flavor compounds in rice wine. LWT-Food Sci. Technol..

[15] Yan Y., Chen H., Sun L., Zhang W., Lu X., Li Z., Xu J., Ren Q. (2022). The changes of microbial diversity and flavor compounds during the fermentation of millet Huangjiu, a traditional Chinese beverage. PLoS ONE.

[16] Dhakal D., Pokhrel A.R., Jha A.K., Thuan N.H., Sohng J.K. (2017). *Saccharopolyspora* species: Laboratory maintenance and enhanced production of secondary metabolites. Curr. Protoc. Microbiol..

[17] Sayed A.M., Abdel--Wahab N.M., Hassan H.M., Abdelmohsen U.R. (2020). *Saccharopolyspora*: An underexplored source for bioactive natural products. J. Appl. Microbiol..

[18] Liu S., Zhang Z.-F., Mao J., Zhou Z., Zhang J., Shen C., Wang S., Marco M.L., Mao J. (2023). Integrated meta-omics approaches reveal Saccharopolyspora as the core functional genus in huangjiu fermentations. npj Biofilms Microbiomes.

[19] Post D.A., Luebke V.E. (2005). Purification, cloning, and properties of a-galactosidase from *Saccharopolyspora erythraea* and its use as a reporter system. Appl. Genet. Mol. Biotechnol..

[20] Yang Y., Xia Y., Wang G., Yu J., Ai L. (2017). Effect of mixed yeast starter on volatile flavor compounds in Chinese rice wine during different brewing stages. LWT.

[21] Liu S., Chen Q., Zou H., Yu Y., Zhou Z., Mao J., Zhang S. (2019). A metagenomic analysis of the relationship between microorganisms and flavor development in Shaoxing mechanized huangjiu fermentation mashes. Int. J. Food Microbiol..

[22] Gao X., Zhao X., Hu F., Fu J., Zhang Z., Liu Z., Wang B., He R., Ma H., Ho C.-T. (2023). The latest advances on soy sauce research in the past decade: Emphasis on the advances in China. Food Res. Int..

[23] Bourbon-Melo N., Palma M., Rocha M.P., Ferreira A., Bronze M.R., Elias H., Sá-Correia I. (2021). Use of Hanseniaspora guilliermondii and Hanseniaspora opuntiae to enhance the aromatic profile of beer in mixed-culture fermentation with *Saccharomyces cerevisiae*. Food Microbiol..

[24] Sumby K.M., Grbin P.R., Jiranek V. (2010). Microbial modulation of aromatic esters in wine: Current knowledge and future prospects. Food Chem..

[25] Swain M.R., Anandharaj M., Ray R.C., Parveen Rani R. (2014). Fermented fruits and vegetables of Asia: A potential source of probiotics. Biotechnol. Res. Int..

[26] Herkenhoff M.E., Battistini C., Praia A.B., Rossini B.C., Dos Santos L.D., Brödel O., Frohme M., Saad S.M.I. (2023). The combination of omics strategies to evaluate starter and probiotic strains in the Catharina sour Brazilian-style beer. Food Res. Int..

[27] Modzelewska A., Jackowski M., Trusek A. (2023). Optimization of beer mixed fermentation using *Saccharomyces cerevisiae* and *Lactobacillus brevis*. Eur. Food Res. Technol..

[28] Virdis C., Sumby K., Bartowsky E., Jiranek V. (2021). Lactic acid bacteria in wine: Technological advances and evaluation of their functional role. Front. Microbiol..

[29] Zhang J., Hou Y., Liu Q., Zhang Y., Gao B., Zou W., Zhang K. (2023). Fortified Jiuqu of the Chinese Baijiu: A review on its functional microorganisms, strengthening effects, current challenges, and perspectives. Food Biosci..

[30] Cha J., Park S.-E., Kim E.-J., Seo S.-H., Cho K.-M., Kwon S.J., Lee M.-H., Son H.-S. (2023). Effects of saccharification agents on the microbial and metabolic profiles of Korean rice wine (makgeolli). Food Res. Int..

[31] Naganuma K., Nakagawa Y., Kokubo S., Hashimoto T., Higuchi K., Ariizumi N., Hayakawa M., Yamamura H. (2023). Traditional microbial control methods used in sake brewing effectively suppress predominant bacteria emerging during production of rice koji. Biotechnol. Biotechnol. Equip..

[32] Xu J., Wu H., Wang Z., Zheng F., Lu X., Li Z., Ren Q. (2018). Microbial dynamics and metabolite changes in Chinese Rice Wine fermentation from sorghum with different tannin content. Sci. Rep..

[33] Chen T., Wu F., Guo J., Ye M., Hu H., Guo J., Liu X. (2020). Effects of glutinous rice protein components on the volatile substances and sensory properties of Chinese rice wine. J. Sci. Food Agric..

[34] Ahmed S., Keniry M., Padilla V., Anaya-Barbosa N., Javed M.N., Gilkerson R., Gomez K., Ashraf A., Narula A.S., Lozano K. (2023). Development of pullulan/chitosan/salvianolic acid ternary fibrous membranes and their potential for chemotherapeutic applications. Int. J. Biol. Macromol..

[35] Area M.R., Rico M., Montero B., Barral L., Bouza R., López J., Ramírez C. (2019). Corn starch plasticized with isosorbide and filled with microcrystalline cellulose: Processing and characterization. Carbohydr. Polym..

[36] Dun H., Liang H., Li S., Li B., Geng F. (2021). Influence of an O/W emulsion on the gelatinization, retrogradation and digestibility of rice starch with varying amylose contents. Food Hydrocoll..

[37] Lu S., Cik T.-T., Lii C.-y., Lai P., Chen H.-H. (2013). Effect of amylose content on structure, texture and α-amylase reactivity of cooked rice. LWT-Food Sci. Technol..

[38] Bai Y., Miao Z., Yan R., Wang X., Cheng Z., Yang J., Wang B., Sun J., Li Z., Zhang Y. (2024). Daqu regulates the balance of saccharification and alcoholic fermentation to promote Chinese baijiu fermentation. Food Biosci..

[39] Gänzle M.G., Follador R. (2012). Metabolism of oligosaccharides and starch in lactobacilli: A review. Front. Microbiol..

[40] Lu Y., Guan X., Li R., Wang J., Liu Y., Ma Y., Lv J., Wang S., Mu J. (2021). Comparative study of microbial communities and volatile profiles during the inoculated and spontaneous fermentation of persimmon wine. Process Biochem..

[41] Shi X., Fan C., Pan C., Zhang F., Hou X., Hui M. (2024). Analysis of differences in physicochemical properties of different sorghum varieties and their influence on the selection of raw materials for winemaking. Food Chem. X.

[42] Yan Y., Liang Z., Huo Y., Wu Q., Ni L., Lv X. (2024). A Comparative Study of Microbial Communities, Biogenic Amines, and Volatile Profiles in the Brewing Process of Rice Wines with Hongqu and Xiaoqu as Fermentation Starters. Foods.

[43] Kurita O., Nakabayashi T., Saitho K. (2003). Isolation and characterization of a high-acetate-producing sake yeast*saccharomyces cerevisiae*. J. Biosci. Bioeng..

[44] Yuan H., Chen W., Chen Y., Wang L., Zhang C., Deng W., Zhang L., Liu G., Shen C., Lou K. (2021). Isolation and characterization of yeast for the production of rice wine with low fusel alcohol content. PLoS ONE.

[45] Yu H., Wang X., Xie J., Ai L., Chen C., Tian H. (2022). Isolation and identification of bitter-tasting peptides in Shaoxing rice wine using ultra-performance liquid chromatography quadrupole time-of-flight mass spectrometry combined with taste orientation strategy. J. Chromatogr. A.

[46] Wang P., Mao J., Meng X., Li X., Liu Y., Feng H. (2014). Changes in flavour characteristics and bacterial diversity during the traditional fermentation of Chinese rice wines from Shaoxing region. Food Control.

[47] Gänzle M.G., Vermeulen N., Vogel R.F. (2007). Carbohydrate, peptide and lipid metabolism of lactic acid bacteria in sourdough. Food Microbiol..

[48] Cocaign-Bousquet M., Garrigues C., Loubiere P., Lindley N.D. (1996). Physiology of pyruvate metabolism in *Lactococcus lactis*. Antonie van Leeuwenhoek.

[49] Leonardi R.J., Racca S., Comelli R.N., Seluy L.G. (2024). Liquid extract with high amino nitrogen obtained by autolysis of brewing yeast can be used as supplement for bioethanol production. Biomass Convers. Biorefin..

[50] Nsogning S.D., Fischer S., Becker T. (2018). Investigating on the fermentation behavior of six lactic acid bacteria strains in barley malt wort reveals limitation in key amino acids and buffer capacity. Food Microbiol..

[51] Zhu Y., Li J., Jiao B., Zhu Q., Zhang X. (2020). Functional microorganisms in tomato stalks/maize straws co-compost unveiled by integrated meta-omics. Fujian J. Agric. Sci..

[52] Zeng J., Sheng F., Hu X., Huang Z., Tian X., Wu Z. (2022). Nutrition promotion of brewer’s spent grain by symbiotic fermentation adding *Bacillus velezensis* and *Levilactobacillus brevis*. Food Biosci..

[53] Zhou C., Zhou Y., Liu T., Li B., Hu Y., Zhai X., Zuo M., Liu S., Yang Z. (2023). Effects of protein components on the chemical composition and sensory properties of millet Huangjiu (Chinese Millet Wine). Foods.

